# Clinical cut-offs for hip- and knee arthroplasty outcome - minimal clinically important improvement (MCII) and patient acceptable symptom state (PASS) of patient-reported outcome measures (PROM)

**DOI:** 10.1007/s11136-025-03896-0

**Published:** 2025-01-20

**Authors:** Aksel Paulsen, Ane Djuv, Ingvild Dalen

**Affiliations:** 1 Department of Orthopedic Surgery, Stavanger University Hospital, Helse Stavanger HF, Stavanger, Norway; 2 The Norwegian Fracture Register, Helse Vest RHF, Stavanger, Norway; 3 Department of Public Health, Faculty of Health Sciences, University of Stavanger, Stavanger, Norway; 4 Department of Clinical Medicine, Faculty of Medicine, University of Bergen, Bergen, Norway; 5 Department of Research, Stavanger University Hospital, Stavanger, Norway

**Keywords:** Arthroplasty, Hip, Knee, Patient reported outcome measures, MCII, PASS.

## Abstract

**Purpose:**

Clinical cut-offs like minimum clinically important improvement (MCII) and patient acceptable symptom state (PASS) increase the interpretability of patient reported outcome measures (PROMs), but cut-off estimates vary considerably, clouding a clear definition of a successful surgical outcome. We report estimates of MCII and PASS following hip- and knee arthroplasty using multiple methods and compare the different estimation methods.

**Methods:**

Elective hip or knee arthroplasty patients who underwent the regular pre- and postoperative assessments 2014–2018 were included. The generic EQ-5D-5L and either the Hip or Knee disability/injury and Osteoarthritis Outcome Score (HOOS/KOOS) were used. MCII and PASS were estimated based on multiple estimation techniques.

**Results:**

Distributions were skewed, with up to 95% being acceptable according to anchor questions. MCII estimates for HOOS/KOOS Pain ranged 21–60/10–47, with fewest in-sample misclassifications for the lowest cut-offs, provided by the 75th percentile approach. PASS estimates for HOOS/KOOS Pain ranged 84–93/78–91, for EQ-5D Index/EQ-VAS 0.87–0.92/66–79 (for hip), and 0.79–0.88/66–76 (for knee), with fewest misclassifications for the 75th percentile approach (hip) and Pythagoras approach (knee). The 75th percentile approach was the approach most often giving MCII estimates below the minimal detectable change (MDC).

**Conclusions:**

We report new one-year estimates of MCII and PASS of HOOS, KOOS and EQ-5D subscales following hip- and knee arthroplasty. Estimates varied considerably when using different anchors and estimation techniques. Overall, the 75th percentile approach had fewest misclassifications, and had the lowest thresholds for the MCII estimations, but which were often below the MDC.

**Supplementary Information:**

The online version contains supplementary material available at 10.1007/s11136-025-03896-0.

## Abstract (summary)

Questionnaires measuring patient outcome following orthopedic surgery are increasingly used, but it is still often difficult to interpret these measures and assess the success of a surgical outcome following hip- and knee arthroplasty. Different methods have been used to better the interpretation and estimate clinically important cut-offs, but the different methods yield dissimilar cut-off estimates, clouding a clear definition of a successful surgical outcome. We have assessed several methods to estimate the clinical important cut-off for two commonly used questionnaires for hip- and knee arthroplasty patients, the Hip disability and Osteoarthritis Outcome Score (HOOS) and the Knee injury and Osteoarthritis Outcome Score (KOOS) and highlight important aspects on how to estimate the clinically important cut-offs. For these joint specific questionnaires with a sum score from 0 (worst) to 100 (best), the minimum clinically important improvement for HOOS and KOOS Pain questions ranged from 21 to 60 (hip) and from 10 to 47 (knee). The postoperative patient acceptable symptom state for the same questions ranged from 84 to 93 (hip) and from 78 to 91 (knee). With our skewed data, where the majority of patients were much improved, we found large differences in MCII estimates using different estimation techniques. Overall, the 75th percentile approach had fewest misclassifications in the material, however this method often resulted in MCII estimates where some changes above the cut-off would be indistinguishable from measurement error.

**Supplementary Information:**

The online version contains supplementary material available at 10.1007/s11136-025-03896-0.

## Background

Patient reported outcome measures (PROMs) are now often routinely used in hip- and knee arthroplasty patients for both clinical utility, research and in arthroplasty registries [1]. Multi-item PROMS may give valid and specific information on relevant domains, for example pain or quality of life, and do give more precise information than single items [2]. Clinical cut-offs like minimum clinically important improvement (MCII) and patient acceptable symptom state (PASS) increase the interpretability of these measures [3], but cut-off estimates vary considerably depending on type of anchors, wording of anchor question and the response options used, and statistical estimation method, clouding a clear definition of a successful surgical outcome.

There is growing awareness that statistical significance should not be equated with clinical relevance, leading to an increased emphasis on reporting effect sizes in research [4]. For PROMs, it can be particularly challenging to determine what constitutes a clinically meaningful difference e.g. between two treatments. A related issue which has high importance in PROM research is identifying what defines a clinically meaningful change within individual patients, and similarly to assess which postoperative PROM scores meet patient expectations for an acceptable state. Establishing such cut-off values is essential to improve the interpretability of PROM results. The Minimal Clinically Important Improvement (MCII) represents the smallest change in a PROM score that patients perceive as a meaningful improvement, reflecting clinically significant progress from their perspective [5]. The PASS is the value of the postoperative PROM score beyond which patients can consider themselves well [6]. Different definitions similar to MCII have been used, i.e. minimum clinically important difference (MCID), minimal important change (MIC), minimal important difference (MID), clinically important responder (CIR), or clinically important difference (CID). The present paper will use MCII to refer to clinically important improvement within an individual [7]. Two main pathways for estimation of MCII have been applied; distribution-based methods and anchor-based methods [8–10]. Distribution-based methods rely only on the statistical characteristics of a group’s PROM scores [11]. A much used metric with regard to MCII is the minimal detectable change (MDC_95_), which is based on the standard error of measurement (SEM) and defined as the threshold equal to and below which the scores of participants drawn from a stable population may be considered to have a 95% probability of no real change having occurred [12]. MDC is equivalent to Reliable change index (RCI) [13], smallest detectable change (SDC) [14], and Coefficient of repeatability [15]. Similar metrics derived from the SEM, such as the sensitivity to change [16] have also been suggested. We will not pursue distribution-based methods in this paper, however, the MDC_95_ will serve as a threshold for evaluating the MCII estimates. If an instrument’s MCII is smaller than its MDC_95_ (for a given population/setting), it means that the measurement error is too high to reliably detect clinically important changes in individual patients [17].

Anchor-based methods see the changes in PROM in relation to changes in an external criterion item. An anchor item is defined as an easily interpretable question which establishes a connection between the PROM change scores (for MCII) or the postoperative PROM scores (for PASS) and the patients’ health situation, thereby increasing the interpretability of the PROM. A current state anchor question may describe, e.g., the patient’s perception of current function after surgery. Another anchor type is the retrospective transition anchor (RTA), in which the patient considers, e.g., the change in function from before to after surgery. The difference in current state anchors at different time-points may also be used as an absolute change anchor [6, 8]. As for PROMs, anchors can focus on general health or be condition specific.

Establishing sound cut-offs for what constitutes a patient reported successful surgical outcome following hip- and knee arthroplasty is important, and MCII and PASS estimates of commonly used PROMs are warranted. It is also warranted to shed light on methodological considerations underlying MCII and PASS estimations. Reported MCII and PASS estimates may serve as reference values for defining successful surgical outcome in both clinical follow-up, outcome studies and registry settings.

MCII and PASS estimates of the included PROMs at 1 year following hip- or knee arthroplasty vary considerably in the literature. MCII HOOS Pain estimates in the area 24–33, MCII HOOS QoL 17–38, MCII EQ-5D Index 0.18–0.41 for HA, and MCII EQ-VAS 12–35 for HA have been reported. For PASS following HA, HOOS Pain estimates lie in the area 75–100, HOOS QoL 50–83, EQ-5D Index 0.77-1.00, and MCII EQ-VAS 72–95 [3, 18]. MCII estimates for KOOS Pain at 6–31, for MCII KOOS QoL at 14 [19, 20]. For PASS following KA, KOOS Pain estimates in the area 77–85, KOOS QoL 63–66, EQ-5D Index 0.75–0.80, and MCII EQ-VAS 70–83 [19, 21]. This considerable variation of cut-offs, and the multitude of methodological approaches, anchors, estimation approaches, and also often incomplete information hindering interpretation of the estimates, renders the cut-offs less clinically usable. Our study adds MCII and PASS estimates using multiple methods, vital information allowing for advanced interpretation of the estimates, including percentage of in-sample misclassification of surgical success for the different estimation approaches. Our study can guide methodological considerations and data publication choices and may help homogenize cut-off estimations to achieve clinically more usable cut-off estimates.

### Aim

The aims of the present study were twofold. First, to add an extensive report of MCII and PASS estimates following hip- and knee arthroplasty in a setting with specialized orthopedic surgery health care in a community characterized by equal rights and high trust, based on real-life high-quality data. Second, by applying a multitude of anchors and reporting and discussing results from multiple estimation methods, to highlight important considerations to be made when estimating cut-offs and when reviewing results from other studies defining successful surgical outcome following hip- and knee arthroplasty.

## Methods

This is a secondary analysis based on previously published data, full details concerning recruitment procedures, subjects, and assessments have been reported elsewhere [22]. Briefly, hip and knee arthroplasty surgery patients from one Norwegian University hospital were included. PROM data were collected preoperative, postoperative at 6 weeks and at one year postoperative. We used the generic EuroQol questionnaire (EQ-5D-5L) and either the Hip or Knee disability/injury and Osteoarthritis Outcome Score (HOOS or KOOS) to measure outcome following hip and knee arthroplasty surgery. The generic health-related quality of life outcome used measures five levels of symptom severity in five dimensions (mobility, self-care, usual activities, pain/discomfort and anxiety/depression) in addition to *current state of health* (EQ-VAS) [23]. HOOS and KOOS is hip/knee specific, include 40/42 items in five dimensions (pain, other symptoms (symptoms), activity/function in daily living (ADL), function in sports and recreation (sports) and joint-related QoL) [24, 25]. Official Norwegian language versions were used. For all our PROMs, higher values coincide with better outcomes; less pain, less symptoms, better function in ADL and sports, better QoL, and better general health-related quality of life.

### Anchors and anchor types

We applied six different anchors with 5-point rating scale response categories (Tables 1 and 2). Anchors like these have previously been examined in the same patient population [1] or are recommended used as best practice [26]. Anchors A2 and A4 represent RTAs, where anchor A2 is joint specific and anchor A4 generic, i.e., relates to general health. Anchors A3 and A5 are current state anchors, generic and joint specific (pain), respectively. Anchors A1 and A6 both relate to the operation results (how would you describe the result/how satisfied are you with the operation), and can be viewed as implicit transition anchors, however maybe with a higher influence of current state than questions explicitly asking the patient to compare with before operation. Responses for the anchor questions were collected at 6 weeks and one year postoperative. We found the best suited anchor based on anchor focus and type (corresponding to the PROM subscale; generic/specific, and current state/retrospective transition) while also taking an adequate anchor-PROM correlation into account. Anchor type was our primary concern (current state anchors for PASS and retrospective transition anchors for MCII), then we looked at anchor focus (corresponding domains, generic/specific), monotonicity in the relationship with PROM scores/changes, and correlation. SDs and reliability estimates used in the MDC estimates, as well as mean PROM scores/changes in PROM scores in relation to anchor categories are shown in Tables 3 and 4 and as supplementary Tables S1-S2, respectively.

**Table 1:** Anchors and anchor-response distributions one year following hip arthroplasty

Anchor	*N*	Response categories and distribution of responses					Acceptable
**A1. ** ***How would you describe the result of the operation?***							
		**1: Excellent**	**2: Very good**	**3: Good**	**4: Fair**	**5: Poor**	**1–3**
	382	155 (40.6%)	137 (35.9%)	61 (16.0%)	21 (5.5%)	8 (2.1%)	353 (92.4%)
**A2. ** ***Overall, how is the operated hip now, compared to before the operation?***							
		**1: Much better**	**2: A little better**	**3: About the same**	**4: A little worse**	**5: Much worse**	**1–2**
	380	320 (84.2%)	42 (11.1%)	8 (2.1%)	7 (1.8%)	3 (0.8%)	362 (95.3%)
**A3. *In general, would you say your health is***:							
		**1: Excellent**	**2: Very good**	**3: Good**	**4: Fair**	**5: Poor**	**1–3**
	378	58 (15.3%)	128 (33.9%)	127 (33.6%)	53 (14.0%)	12 (3.2%)	313 (82.8%)
**A4. ** ***Overall, how is your general health now, compared to before the operation?***							
		**1: Much better**	**2: A little better**	**3: About the same**	**4: A little worse**	**5: Much worse**	**1–2**
	381	254 (66.7%)	67 (17.6%)	42 (11.0%)	12 (3.1%)	6 (1.6%)	321 (84.3%)
**A5. ** ***During the past 4 weeks, how would you describe the pain you usually have in your [right/left] hip?***							
		**1: None**	**2: Very mild**	**3: Mild**	**4: Moderate**	**5: Severe**	**1–3**
	377	235 (62.3%)	61 (16.2%)	41 (10.9%)	34 (9.0%)	6 (1.6%)	337 (89.4%)
**A6. ** ***How satisfied are you with your [right/left] hip replacement?***							
		**1: Very satisfied**	**2: Satisfied**	**3: Neutral**	**4: Dissatisfied**	**5: Very dissatisfied**	**1–2**
	378	242 (64.0%)	93 (24.6%)	11 (2.9%)	16 (4.2%)	16 (4.2%)	335 (88.6%)

**Table 2:** Anchors and anchor-response distributions one year following knee arthroplasty

Anchor	*N*	Response categories and distribution of responses					Acceptable
**A1. ** ***How would you describe the result of the operation?***							
		**1: Excellent**	**2: Very good**	**3: Good**	**4: Fair**	**5: Poor**	**1–3**
	240	70 (29.2%)	89 (37.1%)	45 (18.8%)	31 (12.9%)	5 (2.1%)	204 (85.0%)
**A2. ** ***Overall, how is the operated knee now, compared to before the operation?***							
		**1: Much better**	**2: A little better**	**3: About the same**	**4: A little worse**	**5: Much worse**	**1–2**
	240	193 (80.4%)	26 (10.8%)	12 (5.0%)	4 (1.7%)	5 (2.1%)	219 (91.3%)
**A3. *In general, would you say your health is***:							
		**1: Excellent**	**2: Very good**	**3: Good**	**4: Fair**	**5: Poor**	**1–3**
	240	31 (12.9%)	67 (27.9%)	101 (42.1%)	35 (14.6%)	6 (2.5%)	199 (82.9%)
**A4. ** ***Overall, how is your general health now, compared to before the operation?***							
		**1: Much better**	**2: A little better**	**3: About the same**	**4: A little worse**	**5: Much worse**	**1–2**
	240	138 (57.5%)	43 (17.9%)	38 (15.8%)	14 (5.8%)	7 (2.9%)	181 (75.4%)
**A5. ** ***During the past 4 weeks, how would you describe the pain you usually have in your [right/left] knee?***							
		**1: None**	**2: Very mild**	**3: Mild**	**4: Moderate**	**5: Severe**	**1–3**
	233	98 (42.1%)	62 (26.6%)	28 (12.0%)	37 (15.9%)	8 (3.4%)	188 (80.7%)
**A6. ** ***How satisfied are you with your [right/left] knee replacement?***							
		**1: Very satisfied**	**2: Satisfied**	**3: Neutral**	**4: Dissatisfied**	**5: Very dissatisfied**	**1–2**
	233	130 (55.8%)	64 (27.5%)	18 (7.7%)	15 (6.4%)	6 (2.6%)	194 (83.3%)

### Statistics

Change in PROM was calculated by subtracting the preoperative score from the postoperative score, with a higher change score indicating greater improvement. Assessment of the association of the PROM/change in PROM with anchor questions (see Anchors and anchor types above) was performed using Spearman correlation, and with the dichotomized anchor (acceptable (yes/no) state or change; see details later in this section) using the area under Receiver Operating Characteristics (ROC) curve. Furthermore, mean PROM scores/changes in PROM scores within anchor categories were assessed to consider monotonicity in the relationships. PASS and MCII were estimated by comparison with selected anchors, using the following ROC-based methods: (a) the lowest cut-off achieving 80% specificity for classifying cases as acceptable or not acceptable (specificity refers to the probability of being below the PROM cut-off for patients being deemed non-acceptable on the anchor), (b) the point on the ROC curve closest to intersecting with a -45-degree tangent, (c) the cut-off yielding maximum accuracy, defined as the maximum sum of sensitivity and specificity (i.e., the highest Youden index), and (d) the cut-off corresponding to the smallest sum of squares of (1-sensitivity) and (1-specificity), in accordance with Pythagoras’ theorem [27]; and the following non-ROC-based methods: (e) the mean score/change score among those with anchor responses in the lowest category deemed acceptable, and (f) the 75th percentile method, which for PROMs where higher scores indicate better outcomes, corresponds to the 25th percentile score/change score among the same group as under e). The minimum detectable change at a 95% confidence level (MDC_95_) for each PROM was estimated as MDC_95_ = √ [2] * z * SEM, where z = 1.96 is the 97.5th percentile of the standard normal distribution, and SEM is the standard error of measurement, calculated as SEM = SD * √(1-r), where SD is the standard deviation and r is Cronbach’s alpha of the preoperative PROM scores [28]. Confidence intervals for PASS and MCII estimates were determined through percentile intervals from non-parametric bootstrapping based on B = 1000 resamples. For MCII estimation using non-ROC approaches, only patients with minimal improvement on the anchor were included, while for PASS estimation, patients with good and better anchor states were included. For ROC approaches, dichotomized anchors including all anchor categories deemed acceptable vs. those deemed non-acceptable were used for both MCII and PASS estimation (see Tables 1 and 2 for details of the classification for each anchor). All data analyses were conducted using Stata (StataSE 17.0, StataCorp, 4905 Lakeway Drive, College Station, Texas 77845 USA), employing functions spearman, roctab, rocmic [29], logistic, lsens, and bootstrap.

### Ethics

All methods were performed in accordance with the relevant guidelines and regulations.

The study was submitted for registration to the Regional Committees for Medical and Health Research Ethics (REC), was there considered a follow-up quality-control study under the jurisdiction of the local data protection officer and has been granted an exemption from requiring ethics approval (REC West 2018/268). Due to the use of no personally identifiable data, i.e., only anonymous data reports from patient journals, the study was exempted informed written consent from patients and was approved by the local data protection officer (journal number 6/2018).

## Results

PROM data for 1508 surgical procedures, 917 hip - and 591 knee replacements in 1393 unique patients, was the base of this study. A description of the study population, as well as pre- and postoperative PROM scores and change scores for hip- and knee arthroplasty patients have previously been reported [22].

MCII and PASS estimates are given with 95% confidence intervals in Tables 3, 4, 5 and 6 and varies considerably when using different estimation techniques. Anchor-answer distributions were skewed, with 75–95% being satisfied according to anchor questions (Tables 1 and 2). MCII estimates for HOOS Pain ranged from 21 to 60, for HOOS Symptoms 20–51, for HOOS ADL 13–59, for HOOS Sports 31–63, and for HOOS QoL 14–69 (Table 3). The threshold was lowest in all subscales (except for HOOS ADL; second lowest) for the 75th percentile approach. All subscales had lowest percentage of misclassification for the 75th percentile approach (except for HOOS ADL; second lowest) (Table S5). MCII estimates for KOOS Pain ranged from 10 to 47, for KOOS Symptoms 8–30, for KOOS ADL 14–38, for KOOS Sports 3–31, and for KOOS QoL 19–44 (Table 4). The estimated threshold was lowest in all subscales for the 75th percentile approach. All subscales had lowest percentage of in-sample misclassification for the 75th percentile approach (Table S6). PASS estimates for HOOS Pain ranged from 84 to 93, for HOOS Symptoms 82–90, for HOOS ADL 85–92, for HOOS Sports 63–78, for HOOS QoL 76–88, for EQ-5D Index 0.87–0.92, and for EQ-VAS 66–79 (Table 5). The mean approach and 80% specificity approach often gave the highest thresholds. The 75th percentile approach was among the approaches with lowest percentage of in-sample misclassification for all subscales (except for HOOS Pain; among second lowest), and there was a tendency towards the mean approach having highest percentage of misclassifications (Table S7). PASS estimates for KOOS Pain ranged from 78 to 91, for KOOS Symptoms 80–89, for KOOS ADL 83–89, for KOOS Sports 26–56, for KOOS QoL 63–80, for EQ-5D Index 0.79–0.88, and for EQ-VAS 66–76 (Table 6). The threshold was highest in all subscales for the mean approach. The ROC approaches had lowest percentage of misclassification, and the mean approach had highest percentage of misclassifications for all subscales (Table S8). Different choices for anchors also resulted in dissimilar estimates (data not shown). Mean PROM scores/changes in PROM scores within anchor categories are reported in Tables S1 and S2. Spearman correlations for all anchors and all PROM domains are listed in Tables S3 and S4.

**Table 3:** Minimal clinically important improvement (MCII) for HOOS at one year following hip arthroplasty – multiple estimation methods

	HOOS Pain	HOOS Symptoms	HOOS ADL	HOOS Sports	HOOS QoL
No. of items	10	5	17	4	4
N^1^	682	686	683	674	683
SD	15.4	17.3	16.0	17.6	14.6
Cronbach’s alpha	0.88	0.73	0.94	0.82	0.76
SEM	5.3	9.0	3.9	7.5	7.2
MDC_95_	15 (14, 15)	25 (24, 26)	11 (11, 11)	21 (20, 22)	20 (19, 21)
Anchor used	A2	A2	A2	A4	A2
N^2^	276	279	277	276	279
Spearman’s rho	-0.38	-0.36	-0.33	-0.37	-0.47
N_1_/N_0_^3^	265/11	268/11	266/11	236/40	268/11
AUC	0.71	0.74	0.69	0.74	0.80
**MCII estimates**					
*ROC approaches*					
80% specificity	60 (35, 73)	50 (30, 70)	59 (39, 78)	63 (44, 81)	69 (25, 81)
-45° tangent line	49 (36, 59)	46 (31, 51)	51 (41, 58)	45 (39, 55)	51 (32, 64)
Youden	41 (14, 61)	51 (11, 51)	13 (10, 69)	45 (26, 55)	32 (7, 70)
Pythagoras	41 (24, 51)	31 (16, 51)	51 (10, 51)	45 (32, 55)	51 (14, 60)
*Non-ROC approaches*					
N^4^	32	32	32	31	32
Mean	34 (27, 41)	32 (25, 39)	34 (27, 41)	47 (39, 54)	33 (25, 41)
75th percentile	21 (6, 30)	20 (15, 25)	21 (3, 34)	31 (19, 38)	14 (6, 27)

MDC_95_ and MCII estimates reported with 95% confidence intervals in paratheses. MDC_95_ based on preoperative Cronbach’s alpha. Anchor A2: joint specific retrospective transition anchor; Anchor A4: general (health) retrospective transition anchor

^1^ Number of included cases at baseline (preoperatively), i.e. those with > = 50% valid item responses for the given subscale

^2^ Number of cases that had both the anchor response and a change score for the given subscale, i.e. >=50% valid items responses both at baseline (preoperatively) and at one year postoperatively

^3^ Number of acceptable anchor responses (i.e., “Much better” or “A little better”) / number of not acceptable anchor responses (i.e., “About the same”, “A little worse”, or “A lot worse”) for the subsample under 2)

^4^ Number of included cases that responded “A little better” on the anchor

Abbreviations: HOOS, Hip disability and Osteoarthritis Outcome Score; ADL, Activities of Daily Living; QoL, Quality of Life; SD, Standard deviation; SEM, Standard error of measurement; MDC, Minimal Detectable Change; AUC, Area under (the ROC) curve; ROC, Receiver Operating Characteristics

**Table 4:** Minimal clinically important improvement (MCII) for KOOS at one year following knee arthroplasty – multiple estimation methods

	KOOS Pain	KOOS Symptoms	KOOS ADL	KOOS Sports	KOOS QoL
No. of items	9	7	17	5	4
N^1^	437	443	437	434	446
SD	13.3	13.3	13.2	13.3	13.3
Cronbach’s alpha	0.85	0.69	0.94	0.85	0.66
SEM	5.2	7.4	3.2	7.4	7.8
MDC_95_	18 (17, 18)	28 (27, 29)	12 (11, 12)	18 (16, 20)	21 (20, 23)
Anchor used	A2	A2	A2	A2	A2
N^2^	178	177	174	176	177
Spearman’s rho	-0.44	-0.29^5^	-0.34	-0.33	-0.48
N_1_/N_0_^3^	164/14	164/13	162/12	162/14	163/14
AUC	0.83	0.75	0.81	0.76	0.86
**MCII estimates**					
*ROC approaches*					
80% specificity	47 (14, 81)	29 (18, 75)	37 (24, 63)	30 (10, 85)	44 (13, 69)
-45° tangent line	32 (23, 45)	26 (19, 30)	36 (25, 39)	21 (11, 31)	39 (20, 45)
Youden	32 (12, 48)	30 (1, 37)	38 (7, 41)	31 (1, 31)	26 (14, 45)
Pythagoras	32 (15, 48)	30 (15, 30)	38 (11, 41)	16 (1, 31)	26 (14, 45)
*Non-ROC approaches*					
N^4^	18	18	18	17	17
Mean	26 (16, 36)	23 (13, 33)	33 (24, 42)	24 (12, 37)	30 (22, 38)
75th percentile	10 (-7, 23)	8 (-11, 18)	14 (10, 29)	3 (0, 11)	19 (6, 28)

MDC_95_ and MCII estimates reported with 95% confidence intervals in paratheses. MDC_95_ based on preoperative Cronbach’s alpha. Anchor A2: joint specific retrospective transition anchor

^1^ Number of included cases at baseline (preoperatively), i.e. those with > = 50% valid item responses for the given subscale

^2^ Number of cases that had both the anchor response and a change score for the given subscale, i.e. >=50% valid items responses both at baseline (preoperatively) and at one year postoperatively

^3^ Number of acceptable anchor responses (i.e., “Much better” or “A little better”) / number of not acceptable anchor responses (i.e., “About the same”, “A little worse”, or “A lot worse”) for the subsample under 2)

^4^ Number of included cases that responded “A little better” on the anchor

^5^ Correlation below the 0.30-cut-off

Abbreviations: KOOS, Knee injury and Osteoarthritis Outcome Score; ADL, Activities of Daily Living; QoL, Quality of Life; SD, Standard deviation; SEM, Standard error of measurement; MDC, Minimal Detectable Change; AUC, Area under (the ROC) curve; ROC, Receiver Operating Characteristics

**Table 5:** Patient–acceptable symptom state (PASS) for HOOS and EQ-5D at one year following hip arthroplasty – multiple estimation methods

	HOOS Pain	HOOS Symptoms	HOOS ADL	HOOS Sports	HOOS QoL	EQ-5D Index	EQ VAS
No. of items	10	5	17	4	4	5	1
Anchor used	A5	A5	A5	A1	A5	A3	A3
N^1^	370	372	372	376	372	371	372
Spearman’s rho	-0.66	-0.53	-0.50	-0.49	-0.60	-0.56	-0.65
N_1_/N_0_^2^	332/38	333/39	333/39	347/29	333/39	306/65	307/65
AUC	0.86	0.83	0.81	0.83	0.84	0.80	0.84
**PASS estimates**							
*ROC approaches*							
80% specificity	89 (81, 96)	90 (75, 95)	92 (82, 94)	76 (64, 82)	81 (63, 94)	0.92 (0.86, 0.94)	71 (63, 81)
-45° tangent line	89 (84, 93)	82 (76, 89)	88 (83, 92)	68 (57, 70)	76 (70, 82)	0.88 (0.84, 0.89)	66 (66, 71)
Youden	84 (71, 91)	82 (71, 82)	85 (77, 95)	76 (39, 82)	76 (64, 82)	0.87 (0.79, 0.95)	71 (63, 71)
Pythagoras	89 (81, 91)	82 (71, 82)	85 (79, 93)	64 (51, 76)	76 (64, 82)	0.87 (0.83, 0.92)	71 (63, 71)
*Non-ROC approaches*							
N^3^	332	333	333	347	333	306	307
Mean	93 (92, 94)	90 (89, 91)	91 (89, 92)	78 (76, 81)	88 (86, 90)	0.90 (0.89, 0.92)	79 (77, 81)
75th percentile	90 (88, 93)	85 (83, 85)	87 (84, 90)	63 (63, 69)	81 (78, 88)	0.87 (0.83, 0.88)	70 (70, 75)

PASS estimates reported with 95% confidence intervals in paratheses. Anchor A1: operation results; Anchor A3: general (health) current state anchor; Anchor A5: joint specific (pain) current state anchor. See Table 3 for distributional statistics for the subscales for HOOS, similar measures not available for EQ-5D due to missing preoperative responses

^1^ Number of cases that had both the anchor response and the one-year score for the given subscale, i.e. >=50% valid items responses at one year postoperatively

^2^ Number of acceptable anchor responses / number of not acceptable anchor responses (see Table 1) for the subsample under 1)

^3^ Number of included cases that had acceptable anchor responses

Abbreviations: HOOS, Hip disability and Osteoarthritis Outcome Score; EQ-5D, EuroQol health status questionnaire; ADL, Activities of Daily Living; QoL, Quality of Life; VAS, Visual Analogue Scale; AUC, Area under (the ROC) curve; ROC, Receiver Operating Characteristics

**Table 6:** Patient–acceptable symptom state (PASS) for KOOS and EQ-5D at one year following knee arthroplasty – multiple estimation methods

	KOOS Pain	KOOS Symptoms	KOOS ADL	KOOS Sports	KOOS QoL	EQ-5D Index	EQ VAS
No. of items	9	7	17	5	4	5	1
Anchor used	A5	A5	A5	A5	A5	A5	A3
N^2^	232	232	230	230	231	227	234
Spearman’s rho	-0.78	-0.62	-0.63	-0.47	-0.70	-0.55	-0.60
N_1_/N_0_^3^	187/45	187/45	186/44	186/44	187/44	182/45	194/40
AUC	0.91	0.86	0.86	0.80	0.88	0.82	0.82
**PASS estimates**							
*ROC approaches*							
80% specificity	78 (67, 89)	82 (79, 89)	84 (76, 93)	45 (35, 65)	63 (50, 75)	0.86 (0.79, 0.94)	75 (60, 75)
-45° tangent line	82 (76, 87)	80 (76, 83)	83 (76, 86)	36 (31, 46)	64 (57, 70)	0.83 (0.79, 0.86)	69 (56, 71)
Youden	79 (70, 87)	83 (69, 87)	83 (61, 94)	41 (31, 51)	64 (51, 76)	0.82 (0.70, 0.92)	76 (51, 81)
Pythagoras	79 (76, 82)	80 (76, 83)	83 (66, 85)	41 (31, 46)	64 (51, 70)	0.82 (0.78, 0.86)	66 (51, 76)
*Non-ROC approaches*							
N^4^	187	187	186	186	187	182	194
Mean	91 (89, 92)	89 (87, 90)	89 (87, 90)	57 (53, 60)	80 (77, 83)	0.88 (0.86, 0.90)	76 (73, 79)
75th percentile	86 (83, 89)	82 (79, 86)	84 (78, 87)	35 (30, 45)	69 (63, 75)	0.83 (0.81, 0.86)	67 (60, 70)

PASS estimates reported with 95% confidence intervals in paratheses. Anchor A3: general (health) current state anchor; Anchor A5: joint specific (pain) current state anchor. See Table 4 for distributional statistics for the subscales for KOOS, similar measures not available for EQ-5D due to missing preoperative responses

^1^ Number of cases that had both the anchor response and the one-year score for the given subscale, i.e. >=50% valid items responses at one year postoperatively

^2^ Number of acceptable anchor responses / number of not acceptable anchor responses (see Table 2) for the subsample under 1)

^3^ Number of included cases that had acceptable anchor responses

Abbreviations: KOOS, Knee injury and Osteoarthritis Outcome Score; EQ-5D, EuroQol health status questionnaire; ADL, Activities of Daily Living; QoL, Quality of Life; VAS, Visual Analogue Scale; AUC, Area under (the ROC) curve; ROC, Receiver Operating Characteristics

## Discussion

Our one-year estimates of MCII and PASS of HOOS, KOOS and EQ-5D following hip- and knee arthroplasty varied considerably when using different anchors and estimation techniques. They were also afflicted with high uncertainty, reflected in wide confidence intervals. Comparing estimates across different contexts is anticipated to further increase variation, as it is increasingly recognized that there is no such thing as a single MCII value that is applicable for a PROM across contexts [30, 31]. MCII and PASS varies with, e.g., patient characteristics, type of intervention undergone, and time to follow-up. As Beard et al. state: “*…a cut off level for clinical importance is never an absolute or unquestionable and any results using it need to be interpreted carefully”* [32]. Our results illustrate the importance of accompanying estimates of clinical cut-offs with information on follow-up time, MDC, estimation techniques, anchor wording and distribution, anchor-PROM correlation, AUC (for ROC approaches) as well as uncertainty measures (confidence intervals), as the applicability of the MCII and PASS estimates depends on the setting, and different estimation techniques do not perform equally well when data is skewed. We have not directly studied the impact of patient characteristics, clinical and societal setting and type of intervention undergone, but this information is also important and should be reported together with cut-off estimates. Our focus on the need to report more data than only the estimates of cut-off threshold for clinical importance is in line with the increased focus on standardized PROM reporting [11, 33–36] and the call for greater transparency and consensus in cut-off estimations [7, 20].

We found a larger range of MCII estimates from the different approaches than previously reported for HOOS Pain and HOOS QoL in the same patient group and follow-up time [3], which underlines the effect of anchor wording as shown by Molino et al. [20]. Their proposed threshold of 31 for MCII KOOS Pain is close to our findings. The 75th percentile approach yielded overall lowest MCII estimates, had the lowest percentage of misclassifications, but had more estimates below the MDC than other approaches. For HOOS Pain, HOOS QoL, EQ-5D Index and EQ-VAS, the PASS estimates had a more similar distribution for different approaches [3], all above the 0.77 threshold reported by Florissi et al. [18]. For all KOOS subscales included and EQ-VAS (and EQ-5D-3 L Index), the PASS estimates found were similar to the thresholds reported by Connelly et al. [21]. The 75th percentile approach had fewest overall misclassifications, and for the PASS estimations the mean approach had highest percentage of misclassifications.

As expected, our MCII and PASS estimates had a varying proximity to published estimates for the same PROMs, patient group, and time point [3, 18, 37]. This can be explained by different anchor wording and different estimation techniques. Lyman et al. [38] reported similar HOOS and KOOS MDC_95_ for pain domains, lower for symptoms and QoL, and higher for ADL. Their anchor-based MCII estimates for HOOS was most similar to our non-ROC approaches, except for ADL which was very similar to our result for the Youden approach. For KOOS, our 75th percentile approach gave almost identical results. Importantly, Lyman et al. contrast anchor categories “moderately improved” vs. “no” and “a little improved” in their MCII estimation. Furthermore, different MCII estimates are to be anticipated for different time points, however for most conditions steady state and little improvement will be seen beyond one year postoperatively. Molino et al. reported very similar cut-off for knee arthroplasty patients (KOOS Pain, one year follow-up) as our results using the Youden method (31 vs. 32), but dissimilar results using a low sample size, a very wide definition of change as basis of cut-off estimates, and the VR-12 anchor, which may illustrate the difficulty achieving similar estimates with different anchors, definition of change or sample size [20]. Despite the multiple factors affecting cut-off estimates, the recommended cut-off values by Deckey et al. for HOOS Pain and HOOS QoL (and also median anchor-based values for EQ-5D Index and EQ-VAS) coincidently matches published 75th percentile approach values [3], probably due to low number of included studies, and mean follow-up of 12 months. Pooling different follow-up times when estimating MCII [39] is problematic, as the MCII is dependent on the follow-up time [30].

### Different estimation techniques

It is not possible to define what is clinically important by using distribution-based methods [40]. To be able to estimate cut-offs based on the patients’ perspective, anchor items are imperative and anchor-based methods should be used, with distribution-based approaches providing supportive information [1, 3]. The mean change approach and the 75th percentile approach are common and simple anchor-based approaches, but by definition the mean change approach misclassifies about half of the patients that are deemed just acceptable on the anchor [34], and the 75th percentile approach misclassifies a quarter of the same group. All ROC approaches, except the 80% specificity approach, value sensitivity and specificity equally [27]. Among methods that were not tested in the present study, item response theory-based methods may represent an improvement being less sensitive to anchor distributions than ROC-based methods, but has currently only been tested in situations with a true gold standard [41].

### Anchor-PROM correlation

An adequate anchor-PROM correlation is important, but recommendations vary from at least 0.30, between 0.40 and 0.70, to over 0.50 in absolute value [8, 36, 42]. Using a poorly related anchor can lead to cut-offs that are contaminated with noise and are of little or no use [36]. It is strongly recommended to use multiple anchors [43]. Given this, presentation of anchor-PROM correlations is important as context for evaluating published cut-off estimates [44]. Only one of our correlations fall below 0.30 in absolute value (MCII for HOOS Symptoms) however none of the correlations for change (MCII) are above 0.50. The anchor-PROM correlations are all within 0.40–0.70, for the PASS estimates, except for KOOS Pain (-0.78). It is uncertain if the higher correlations for non-RTAs in Tables S3 and S4 were due to domain (we have no retrospective transition anchor on pain), recall bias/present state bias or due to other reasons.

### Minimal detectable change

Our MDC_95_ estimates were based on preoperative standard deviation and Cronbach alpha, as the preoperative samples were biggest (*n* = 674–686 for HOOS, *n* = 434–446 for KOOS). Ideally, it should have been based on test-retest reliability instead of internal consistency, however such data were not available to us. The test-retest reliability may be poorer than the internal consistency, which may lead to a higher MDC. Our MDC for HOOS Pain is identical to previous findings and almost identical (20 vs. 21) for HOOS QoL [1]. The MCII cut-off estimate from the 75th percentile approach was lower than the MDC for HOOS Symptoms, HOOS QoL and all KOOS subscales except KOOS ADL. For these, the difference was highest for KOOS Symptoms – this was also the MCII estimate that was based on an anchor with correlation < 0.30 with the PROM. An MCII lower than the MDC of an instrument may raise questions regarding the PROM’s utility for detecting the smallest clinically relevant changes in individual patients, however in our case it must also be seen in light of uncertainty regarding the MCII estimates and the wide confidence intervals. In clinical practice, where identifying patients who did not improve for further follow-up is more important, using a higher cut-off (> MDC) would tie up more resources but may ensure that fewer patients with significant needs are overlooked.

### Retrospective transition anchor, recall bias/present state bias and current-state anchors

Recall bias may be a problem with RTAs [8], as well as present state bias [45]. RTAs should have an equal correlation to preoperative- as to postoperative PROMs but may often have a stronger correlation to postoperative PROMs. Repeated current-state anchors may therefore be better than RTAs in this respect. Repeated current-state anchors give information for the specified time point (which can be used for PASS estimation), but also information on absolute change when examining shift in answer category over time, and thus may be used for MCII estimation as well [6]. RTAs on the other hand, may seldom be used for PASS estimation, as these anchors give little information on present status. We were not able to use repeated current-state anchors, due to missing data on preoperative anchors. Using different anchor types and using both RTAs and repeated current-state anchors may pose a solution for minimizing the effect of recall bias.

### Minimal clinically important change?

What constitutes a minimal clinically relevant improvement depends on the nature of the intervention. A small change may be deemed clinically relevant if the intervention has low cost and low risk. The setting of the intervention, i.e., elective or following trauma, as well as patient expectations, may also influence what qualifies as a minimal clinically relevant change. Condition specific outcome and general health outcomes may also be perceived differently. Arthroplasty surgery has high cost and substantial risks and is furthermore well-known to be effective. It is therefore not surprising that only a substantial improvement is considered clinically relevant for these patients. De Vet et al. argue for using «much improved» as a cut-off for acceptable outcome on transition anchors [46]. On the other hand, basing the MCII on large improvements on the anchor may reduce the clinical utility of the cut-off, and it would perhaps no longer be the *minimal* clinically important change. We opted to use those reporting a small positive change on the anchor as basis for estimation of MCII for the non-ROC approaches. On the other hand, corresponding estimation of PASS, where an *acceptable* symptom state is the focus (i.e., not “minimal” acceptable symptom state), was performed with all patients reporting an acceptable state on the anchor.

### Limitations and strengths

Several methodological limitations should be taken into consideration when interpreting the results of the present study. The distribution was skewed, and the dichotomized anchor questions were unbalanced, which together with the limited sample sizes resulted in high degree of uncertainty in our ROC-based cut-off estimates. We did not have test-retest data, thus MDC estimates may be less accurate. Most of the PROM-anchor correlations for the anchors used for MCII were on the low side. We did not have pre-operative anchors, thus had to use retrospective change anchors for MCII. Few patients (< 100 in each group) answered the preoperative EQ-5D questionnaire, these data as well as MCII estimates for EQ-5D are not presented due to low sample size. Misclassification was assessed in-sample. Strengths: The response rates for all PROMs were high (≥ 80%). High response rates ensure generalizability and minimize selection bias, and a response rate ≥ 80% is usually considered to be adequate and sufficiently representative of the sample studied [47]. We used well-validated PROMs for the patient group, with validated feasibility [48]. Standardized format paper PROMs were used. All returned questionnaire forms were scanned electronically using a validated automated form-processing technique [49]. Although not a limitation specifically for our study, cut-off estimates should not be used for between-group comparisons of mean outcome scores, but for defining individual successful surgical outcome following hip- and knee arthroplasty [50].

## Conclusions

We have reported one-year estimates of MCII and PASS of HOOS, KOOS and EQ-5D following hip- and knee arthroplasty. MCII and PASS estimates vary considerably when using different anchors and estimation techniques. Different estimation techniques do not perform equal when the data material is skewed, and the 75th percentile approach had overall fewest misclassifications in our material. The 75th percentile approach also had the lowest threshold for the MCII estimations, often below the MDC.

## Electronic supplementary material

Below is the link to the electronic supplementary material.

Supplementary Material 1: **Table S1.** Anchors and anchor-response distributions one year following hip arthroplasty, with mean PROM scores/changes within response categories. **Table S2.** Anchors and anchor-response distributions one year following knee arthroplasty, with mean PROM scores/changes within response categories. **Table S3.** Spearman correlations between anchor and change in PROM score. **Table S4.** Spearman correlations between anchor and PROM score. **Table S5.** Total in-sample misclassification (%) for different estimates of Minimal clinically important improvement (MCII) cut-offs for HOOS one year following hip arthroplasty, with gold standard given by acceptable/non-acceptable changes on the anchor used. **Table S6.** Total in-sample misclassification (%) for different estimates of Minimal clinically important improvement (MCII) cut-offs for KOOS domains one year following knee arthroplasty, with gold standard given by acceptable/non-acceptable changes on the anchor used. **Table S7.** Total in-sample misclassification (%) for different estimates of Patient-acceptable symptoms state (PASS) cut-offs for HOOS domains and EQ-5D Index and VAS one year following hip arthroplasty, with gold standard given by acceptable/non-acceptable states for the anchor used. **Table S8**. Total in-sample misclassification (%) for different estimates of Patient-acceptable symptoms state (PASS) cut-offs for KOOS domains and EQ-5D Index and VAS one year following knee arthroplasty, with gold standard given by acceptable/non-acceptable states for the anchor used

## Data Availability

All data generated or analyzed during this study are included in this published article [and its supplementary information files].

## Abbreviations

ADL: Activity in Daily Living
AUC: Area Under the Curve
CI: Confidence Intervals
CID: Clinically Important Difference
CIR: Clinically Important Responder
EQ-5D: EuroQol questionnaire
HA: Hip Arthroplasty
HOOS: Hip disability and Osteoarthritis Outcome Score
KA: Knee Arthroplasty
KOOS: Knee injury and Osteoarthritis Outcome Score
MCID: Minimum Clinically Important Difference
MCII: Minimal Clinically Important Improvement
MDC: Minimal detectable change
MIC: Minimal Important Change
MID: Minimal Important Difference
PASS: Patient Acceptable Symptom State
PROM: Patient-Reported Outcome Measures
QoL: Quality of Life
REC: Regional Committees for Medical and Health Research Ethics
ROC: Receiver Operating Characteristic
RTA: Retrospective Transition Anchor
SD: Standard Deviations
VAS: Visual Analogue Scale
