# Supplementary material for: Clinical cut-offs for hip- and knee arthroplasty outcome - minimal clinically important improvement (MCII) and patient acceptable symptom state (PASS) of patient-reported outcome measures (PROM)
Source: Qual Life Res. 2025 Jan 20;34(4):1147–58. doi: 10.1007/s11136-025-03896-0 (PMC11982088; doi:10.1007/s11136-025-03896-0)
Supplement: Supplementary file 1 — Supplementary Material 1: Table S1. Anchors and anchor-response distributions one year following hip arthroplasty, with mean PROM scores/changes within response categories. Table S2. Anchors and anchor-response distributions one year following knee arthroplasty, with mean PROM scores/changes within response categories. Table S3. Spearman correlations between anchor and change in PROM score. Table S4. Spearman correlations between anchor and PROM score. Table S5. Total in-sample misclassification (%) for different estimates of Minimal clinically important improvement (MCII) cut-offs for HOOS one year following hip arthroplasty, with gold standard given by acceptable/non-acceptable changes on the anchor used. Table S6. Total in-sample misclassification (%) for different estimates of Minimal clinically important improvement (MCII) cut-offs for KOOS domains one year following knee arthroplasty, with gold standard given by acceptable/non-acceptable changes on the anchor used. Table S7. Total in-sample misclassification (%) for different estimates of Patient-acceptable symptoms state (PASS) cut-offs for HOOS domains and EQ-5D Index and VAS one year following hip arthroplasty, with gold standard given by acceptable/non-acceptable states for the anchor used. Table S8. Total in-sample misclassification (%) for different estimates of Patient-acceptable symptoms state (PASS) cut-offs for KOOS domains and EQ-5D Index and VAS one year following knee arthroplasty, with gold standard given by acceptable/non-acceptable states for the anchor used [file 11136_2025_3896_MOESM1_ESM.docx]

**Supplemental material**

Table S1. Anchors and anchor-response distributions one year following hip arthroplasty, with mean PROM scores/changes within response categories.

| **Anchor/PROM** | **N** | **Anchor categories** | | | | | **Acceptable** | **Not acceptable** |
| --- | --- | --- | --- | --- | --- | --- | --- | --- |
| **A1. *How would you describe the result of the operation?*** | | | | | | |  |  |
|  | | | | | | |  |  |
| *Response distribution* |  | **1: Excellent** | **2: Very good** | **3: Good** | **4: Fair** | **5: Poor** | **1-3** | **4-5** |
|  | 381^1^ | 154 (40.4%) | 137 (36.0%) | 61 (16.0%) | 21 (5.5%) | 8 (2.1%) | 352 (92.4%) | 29 (7.6%) |
| HOOS Pain | 375 | 97.0 | 91.0 | 79.7 | 77.6 | 51.8 | 91.7 | 71.2 |
| HOOS Symptoms | 377 | 94.3 | 87.2 | 79.7 | 72.7 | 55.6 | 89.0 | 68.0 |
| HOOS ADL | 377 | 94.8 | 89.4 | 79.3 | 76.0 | 57.0 | 90.1 | 70.7 |
| HOOS Sports | 376 | 87.0 | 75.7 | 62.0 | 50.4 | 32.7 | 78.3 | 46.6 |
| HOOS QoL | 377 | 95.5 | 86.1 | 72.5 | 51.0 | 36.7 | 87.9 | 47.1 |
| EQ-5D Index | 375 | 0.91 | 0.88 | 0.82 | 0.74 | 0.61 | 0.89 | 0.71 |
| EQ-VAS | 376 | 79.5 | 75.1 | 68.6 | 66.3 | 56.3 | 75.9 | 63.6 |
|  |  | **1: Excellent** | **2: Very good** | **3: Good** | **4: Fair** | **5: Poor** | **1-3** | **4-5** |
| *Response distribution* | 280^1^ | 117 (41.8%) | 100 (35.7%) | 42 (15.0%) | 15 (5.4%) | 6 (2.1%) | 259 (92.5%) | 21 (7.5%) |
| ΔHOOS Pain | 277 | 61.7 | 54.0 | 42.4 | 43.0 | 28.2 | 55.6 | 38.8 |
| ΔHOOS Symptoms | 280 | 58.3 | 51.1 | 41.5 | 33.5 | 21.7 | 52.8 | 30.1 |
| ΔHOOS ADL | 278 | 58.7 | 51.7 | 43.5 | 37.9 | 17.6 | 53.5 | 31.8 |
| ΔHOOS Sports | 277 | 69.1 | 52.9 | 39.7 | 27.6 | 9.0 | 58.0 | 22.3 |
| ΔHOOS QoL | 280 | 75.9 | 64.2 | 46.8 | 29.3 | 16.7 | 66.7 | 25.7 |
|  |  |  |  |  |  |  |  |  |
| **A2. *Overall, how is the operated hip now, compared to before the operation?*** | | | | | | |  |  |
|  | | | | | | |  |  |
| *Response distribution* |  | **1: Much better** | **2: A little better** | **3: About the same** | **4: A little worse** | **5: Much worse** | **1-2** | **3-5** |
|  | 279^1^ | 236 (84.6%) | 32 (11.5%) | 4 (1.4%) | 4 (1.4%) | 3 (1.1%) | 268 (96.1%) | 11 (3.9%) |
| ΔHOOS Pain | 276 | 57.9 | 33.8 | 48.8 | 44.8 | 13.3 | 55.0 | 37.7 |
| ΔHOOS Symptoms | 279 | 54.6 | 32.1 | 43.8 | 38.8 | 5.4 | 51.9 | 31.5 |
| ΔHOOS ADL | 277 | 55.2 | 34.3 | 53.6 | 33.1 | 5.3 | 52.7 | 33.0 |
| ΔHOOS Sports | 276 | 54.5 | 31.0 | 71.9 | 20.3 | 2.1 | 56.1 | 34.1 |
| ΔHOOS QoL | 279 | 69.4 | 32.8 | 57.8 | 21.9 | -6.3 | 65.0 | 27.3 |
|  |  |  |  |  |  |  |  |  |
| **A3. *In general, would you say your health is:*** | | | | | | |  |  |
|  | | | | | | |  |  |
| *Response distribution* |  | **1: Excellent** | **2: Very good** | **3: Good** | **4: Fair** | **5: Poor** | **1-3** | **4-5** |
|  | 377^1^ | 58 (15.4%) | 127 (33.7%) | 127 (33.7%) | 53 (14.1%) | 12 (3.2%) | 312 (82.8%) | 65 (17.2%) |
| HOOS Pain | 371 | 98.1 | 92.5 | 88.3 | 83.6 | 77.7 | 91.8 | 82.5 |
| HOOS Symptoms | 373 | 96.6 | 89.0 | 84.5 | 82.9 | 78.0 | 88.6 | 82.0 |
| HOOS ADL | 373 | 97.3 | 92.3 | 85.8 | 80.1 | 73.0 | 90.6 | 78.7 |
| HOOS Sports | 372 | 92.0 | 82.9 | 69.5 | 61.9 | 46.7 | 79.2 | 59.0 |
| HOOS QoL | 373 | 96.4 | 88.7 | 80.6 | 78.2 | 60.9 | 86.8 | 75.0 |
| EQ-5D Index | 371 | 0.97 | 0.94 | 0.84 | 0.75 | 0.52 | 0.90 | 0.71 |
| EQ-VAS | 372 | 92.0 | 81.4 | 70.1 | 59.2 | 40.4 | 78.9 | 55.7 |
|  |  |  |  |  |  |  |  |  |
| **A4. *Overall, how is your general health now, compared to before the operation?*** | | | | | | |  |  |
|  | | | | | | |  |  |
| *Response distribution* |  | **1: Much better** | **2: A little better** | **3: About the same** | **4: A little worse** | **5: Much worse** | **1-2** | **3-5** |
|  | 279^1^ | 190 (68.1%) | 47 (16.9%) | 29 (10.4%) | 8 (2.9%) | 5 (1.8%) | 237 (85.0%) | 42 (15.1%) |
| ΔHOOS Pain | 276 | 57.8 | 49.6 | 43.2 | 51.4 | 37.5 | 56.1 | 44.1 |
| ΔHOOS Symptoms | 279 | 56.0 | 42.5 | 40.9 | 41.4 | 22.0 | 53.3 | 38.7 |
| ΔHOOS ADL | 277 | 56.3 | 46.8 | 38.5 | 44.9 | 26.2 | 54.4 | 38.2 |
| ΔHOOS Sports | 276 | 62.1 | 46.6 | 39.6 | 23.2 | 15.6 | 59.1 | 34.3 |
| ΔHOOS QoL | 279 | 69.0 | 55.1 | 52.1 | 43.0 | 33.8 | 66.3 | 48.2 |
|  |  |  |  |  |  |  |  |  |
| **A5. *During the past 4 weeks, how would you describe the pain you usually have in your [right/left] hip?*** | | | | | | |  |  |
|  | | | | | | |  |  |
| *Response distribution* |  | **1: None** | **2: Very mild** | **3: Mild** | **4: Moderate** | **5: Severe** | **1-3** | **4-5** |
|  | 376^1^ | 234 (62.2%) | 61 (16.2%) | 41 (10.9%) | 34 (9.0%) | 6 (1.6%) | 336 (89.4%) | 40 (10.6%) |
| HOOS Pain | 370 | 97.0 | 87.2 | 80.2 | 70.5 | 41.3 | 93.1 | 65.9 |
| HOOS Symptoms | 372 | 93.4 | 83.1 | 80.3 | 70.5 | 50.8 | 89.9 | 67.5 |
| HOOS ADL | 372 | 94.1 | 84.3 | 82.1 | 74.3 | 47.3 | 90.8 | 70.1 |
| HOOS Sports | 371 | 81.6 | 71.4 | 68.0 | 62.7 | 26.0 | 78.0 | 57.1 |
| HOOS QoL | 372 | 93.8 | 80.6 | 67.4 | 60.4 | 39.6 | 88.2 | 57.2 |
| EQ-5D Index | 370 | 0.91 | 0.86 | 0.84 | 0.76 | 0.46 | 0.89 | 0.73 |
| EQ-VAS | 371 | 77.3 | 74.2 | 72.8 | 66.0 | 51.0 | 76.2 | 64.1 |
|  |  |  |  |  |  |  |  |  |
| **A6. *How satisfied are you with your [right/left] hip replacement?^2^*** | | | | | | |  |  |
|  | | | | | | |  |  |
| *Response distribution* |  | **1: Very satisfied** | **2: Satisfied** | **3: Neutral** | **4: Dissatisfied** | **5: Very dissatisfied** | **1-2** | **3-5** |
|  | 377^1^ | 241 (63.9%) | 93 (24.7%) | 11 (2.9%) | 16 (4.2%) | 16 (4.2%) | 334 (88.6%) | 43 (11.4%) |
| HOOS Pain | 371 | 95.8 | 81.8 | 74.0 | 71.0 | 85.0 | 91.9 | 77.0 |
| HOOS Symptoms | 373 | 92.6 | 80.3 | 64.1 | 71.8 | 84.5 | 89.2 | 74.8 |
| HOOS ADL | 373 | 93.9 | 80.2 | 76.3 | 73.8 | 79.9 | 90.1 | 76.7 |
| HOOS Sports | 372 | 83.2 | 64.8 | 54.4 | 53.6 | 65.2 | 78.1 | 58.2 |
| HOOS QoL | 373 | 92.7 | 73.7 | 63.8 | 50.4 | 80.1 | 87.4 | 64.9 |
| EQ-5D Index | 371 | 0.91 | 0.83 | 0.75 | 0.70 | 0.81 | 0.89 | 0.75 |
| EQ-VAS | 372 | 78.4 | 67.5 | 71.8 | 66.2 | 76.4 | 75.4 | 71.5 |
|  |  | **1: Very satisfied** | **2: Satisfied** | **3: Neutral** | **4: Dissatisfied** | **5: Very dissatisfied** | **1-2** | **3-5** |
| *Response distribution* | 278^1^ | 175 (63.0%) | 72 (25.9%) | 8 (2.9%) | 8 (2.9%) | 15 (5.4%) | 247 (88.9%) | 31 (11.2%) |
| ΔHOOS Pain | 275 | 61.1 | 43.5 | 42.5 | 38.6 | 44.7 | 55.9 | 42.5 |
| ΔHOOS Symptoms | 278 | 57.3 | 42.7 | 30.2 | 33.8 | 43.8 | 53.0 | 37.7 |
| ΔHOOS ADL | 276 | 58.2 | 42.7 | 41.1 | 36.3 | 40.1 | 53.6 | 39.3 |
| ΔHOOS Sports | 275 | 64.1 | 41.9 | 38.3 | 29.7 | 43.2 | 57.7 | 38.4 |
| ΔHOOS QoL | 278 | 73.0 | 50.6 | 39.1 | 22.7 | 54.6 | 66.4 | 42.3 |
|  |  |  |  |  |  |  |  |  |

^1^ Number of anchor responses that have at least one valid observation among the PROM scores/changes listed and evaluated after. ^2^ Response categories were presented in reverse order. Abbreviations: HOOS, Hip disability and Osteoarthritis Outcome Score; PROM, Patient Reported Outcome Measure; ADL, Activities of Daily Living; QoL, Quality of Life; VAS, Visual analogue scale; Δ (delta), “change in”.

Table S2. Anchors and anchor-response distributions one year following knee arthroplasty, with mean PROM scores/changes within response categories.

| **Anchor/PROM** | **N** | **Anchor categories** | | | | | **Acceptable** | **Not acceptable** |
| --- | --- | --- | --- | --- | --- | --- | --- | --- |
| **A1. *How would you describe the result of the operation?*** | | | | | | |  |  |
|  | | | | | | |  |  |
| *Response distribution* |  | **1: Excellent** | **2: Very good** | **3: Good** | **4: Fair** | **5: Poor** | **1-3** | **4-5** |
|  | 239^1^ | 70 (29.3%) | 88 (36.8%) | 45 (18.8%) | 31 (13.0%) | 5 (2.1%) | 203 (84.9%) | 29 (15.1%) |
| KOOS Pain | 238 | 95.6 | 90.1 | 76.5 | 58.5 | 42.2 | 89.0 | 56.2 |
| KOOS Symptoms | 238 | 92.0 | 88.3 | 77.4 | 69.4 | 55.0 | 87.1 | 67.4 |
| KOOS ADL | 236 | 94.2 | 88.3 | 74.2 | 63.1 | 47.9 | 87.2 | 61.0 |
| KOOS Sports | 236 | 64.4 | 56.5 | 38.3 | 27.5 | 15.0 | 55.2 | 25.7 |
| KOOS QoL | 237 | 89.0 | 79.6 | 59.6 | 44.8 | 28.8 | 78.4 | 42.5 |
| EQ-5D Index | 234 | 0.92 | 0.87 | 0.81 | 0.69 | 0.60 | 0.87 | 0.68 |
| EQ-VAS | 234 | 77.3 | 76.6 | 66.9 | 58.1 | 65.0 | 74.7 | 59.0 |
|  |  | **1: Excellent** | **2: Very good** | **3: Good** | **4: Fair** | **5: Poor** | **1-3** | **4-5** |
| *Response distribution* | 178^1^ | 58 (32.6%) | 65 (36.5%) | 28 (15.7%) | 23 (12.9%) | 4 (2.3%) | 151 (84.8%) | 27 (15.2%) |
| ΔKOOS Pain | 178 | 57.2 | 48.3 | 39.9 | 21.2 | 9.0 | 50.2 | 19.4 |
| ΔKOOS Symptoms | 177 | 42.1 | 37.0 | 22.8 | 22.1 | 9.8 | 36.3 | 20.2 |
| ΔKOOS ADL | 174 | 52.0 | 43.1 | 36.0 | 28.6 | 14.7 | 45.2 | 26.4 |
| ΔKOOS Sports | 176 | 48.8 | 44.5 | 29.1 | 13.6 | 6.3 | 43.3 | 12.5 |
| ΔKOOS QoL | 177 | 69.8 | 55.6 | 39.3 | 25.9 | 3.1 | 58.0 | 22.4 |
|  |  |  |  |  |  |  |  |  |
| **A2. *Overall, how is the operated knee now, compared to before the operation?*** | | | | | | |  |  |
|  | | | | | | |  |  |
| *Response distribution* |  | **1: Much better** | **2: A little better** | **3: About the same** | **4: A little worse** | **5: Much worse** | **1-2** | **3-5** |
|  | 178^1^ | 146 (82.0%) | 18 (10.1%) | 8 (4.5%) | 2 (1.1%) | 4 (2.3%) | 164 (92.1%) | 14 (7.9%) |
| ΔKOOS Pain | 178 | 50.7 | 25.9 | 26.7 | 15.3 | -2.8 | 48.0 | 16.6 |
| ΔKOOS Symptoms | 177 | 37.0 | 23.0 | 21.9 | 26.8 | -4.5 | 35.5 | 14.6 |
| ΔKOOS ADL | 174 | 45.6 | 32.7 | 24.8 | 35.3 | 7.7 | 44.1 | 20.0 |
| ΔKOOS Sports | 176 | 43.2 | 23.5 | 26.9 | 12.5 | -22.5 | 41.1 | 10.7 |
| ΔKOOS QoL | 177 | 58.7 | 30.1 | 28.9 | 28.1 | -7.8 | 55.8 | 18.3 |
|  |  |  |  |  |  |  |  |  |
| **A3. *In general, would you say your health is:*** | | | | | | |  |  |
|  | | | | | | |  |  |
| *Response distribution* |  | **1: Excellent** | **2: Very good** | **3: Good** | **4: Fair** | **5: Poor** | **1-3** | **4-5** |
|  | 239^1^ | 31 (13.0%) | 66 (27.6%) | 101 (42.3%) | 35 (14.6%) | 6 (2.5%) | 198 (82.9%) | 41 (17.2%) |
| KOOS Pain | 238 | 92.6 | 89.4 | 82.5 | 73.1 | 70.8 | 86.4 | 72.8 |
| KOOS Symptoms | 238 | 88.3 | 86.0 | 84.3 | 78.2 | 73.8 | 85.5 | 77.6 |
| KOOS ADL | 236 | 93.9 | 90.8 | 81.2 | 68.2 | 69.6 | 86.5 | 68.4 |
| KOOS Sports | 236 | 64.5 | 60.6 | 48.4 | 31.9 | 27.5 | 55.0 | 31.2 |
| KOOS QoL | 237 | 86.3 | 82.3 | 70.9 | 53.4 | 56.3 | 77.1 | 53.8 |
| EQ-5D Index | 234 | 0.94 | 0.92 | 0.82 | 0.70 | 0.70 | 0.87 | 0.70 |
| EQ-VAS | 234 | 79.6 | 87.0 | 67.7 | 56.2 | 41.7 | 76.0 | 54.0 |
|  |  |  |  |  |  |  |  |  |

| **A4. *Overall, how is your general health now, compared to before the operation?*** | | | | | | |  |  |
| --- | --- | --- | --- | --- | --- | --- | --- | --- |
|  | | | | | | |  |  |
| *Response distribution* |  | **1: Much better** | **2: A little better** | **3: About the same** | **4: A little worse** | **5: Much worse** | **1-2** | **3-5** |
|  | 178^1^ | 110 (61.8%) | 31 (17.4%) | 25 (14.0%) | 7 (3.9%) | 5 (2.8%) | 141 (79.2%) | 37 (20.8%) |
| ΔKOOS Pain | 178 | 50.4 | 39.9 | 41.5 | 29.4 | 14.2 | 48.1 | 35.5 |
| ΔKOOS Symptoms | 177 | 37.8 | 27.1 | 32.9 | 24.0 | 10.7 | 35.5 | 28.1 |
| ΔKOOS ADL | 174 | 46.5 | 35.7 | 39.9 | 37.4 | 11.5 | 44.1 | 36.2 |
| ΔKOOS Sports | 176 | 46.2 | 27.3 | 33.0 | 11.7 | 7.0 | 42.0 | 25.8 |
| ΔKOOS QoL | 177 | 60.3 | 44.0 | 25.5 | 21.3 | 37.9 | 56.7 | 37.5 |
|  |  |  |  |  |  |  |  |  |
| **A5. *During the past 4 weeks, how would you describe the pain you usually have in your [right/left] knee?*** | | | | | | |  |  |
|  | | | | | | |  |  |
| *Response distribution* |  | **1: None** | **2: Very mild** | **3: Mild** | **4: Moderate** | **5: Severe** | **1-3** | **4-5** |
|  | 232^1^ | 98 (42.2%) | 61 (26.3%) | 28 (12.1%) | 37 (16.0%) | 8 (3.5%) | 187 (80.6%) | 45 (19.4%) |
| KOOS Pain | 232 | 96.9 | 87.7 | 76.4 | 63.1 | 34.2 | 90.8 | 58.0 |
| KOOS Symptoms | 232 | 93.0 | 86.8 | 77.9 | 69.2 | 53.6 | 88.7 | 66.4 |
| KOOS ADL | 230 | 94.2 | 85.0 | 76.8 | 65.8 | 40.9 | 88.6 | 61.8 |
| KOOS Sports | 230 | 63.0 | 55.0 | 38.0 | 30.0 | 10.7 | 56.7 | 26.9 |
| KOOS QoL | 231 | 89.2 | 74.5 | 60.7 | 48.0 | 28.6 | 80.1 | 44.9 |
| EQ-5D Index | 227 | 0.93 | 0.84 | 0.83 | 0.74 | 0.43 | 0.88 | 0.68 |
| EQ-VAS | 227 | 77.2 | 72.4 | 72.9 | 62.0 | 55.6 | 75.0 | 60.9 |
|  |  |  |  |  |  |  |  |  |
| **A6. *How satisfied are you with your [right/left] knee replacement?^2^*** | | | | | | |  |  |
|  | | | | | | |  |  |
| *Response distribution* |  | **1: Very satisfied** | **2: Satisfied** | **3: Neutral** | **4: Dissatisfied** | **5: Very dissatisfied** | **1-2** | **3-5** |
|  | 232^1^ | 130 (56.0%) | 63 (27.2%) | 18 (7.8%) | 15 (6.5%) | 6 (2.6%) | 193 (83.2%) | 39 (16.8%) |
| KOOS Pain | 232 | 93.8 | 81.9 | 54.8 | 55.0 | 74.5 | 89.9 | 57.9 |
| KOOS Symptoms | 232 | 91.6 | 80.4 | 67.8 | 63.7 | 77.4 | 88.0 | 67.7 |
| KOOS ADL | 230 | 93.7 | 76.1 | 59.9 | 58.9 | 73.3 | 87.9 | 61.6 |
| KOOS Sports | 230 | 63.5 | 39.9 | 22.4 | 26.1 | 47.5 | 55.8 | 27.8 |
| KOOS QoL | 231 | 86.8 | 64.0 | 42.0 | 38.4 | 63.5 | 79.3 | 44.1 |
| EQ-5D Index | 227 | 0.92 | 0.80 | 0.72 | 0.64 | 0.68 | 0.88 | 0.68 |
| EQ-VAS | 227 | 77.5 | 71.1 | 58.5 | 58.3 | 45.0 | 75.4 | 56.3 |
|  |  | **1: Very satisfied** | **2: Satisfied** | **3: Neutral** | **4: Dissatisfied** | **5: Very dissatisfied** | **1-2** | **3-5** |
| *Response distribution* | 174^1^ | 99 (56.9%) | 47 (27.0%) | 13 (7.5%) | 10 (5.8%) | 5 (2.9%) | 146 (83.9%) | 28 (16.1%) |
| ΔKOOS Pain | 174 | 54.3 | 42.3 | 23.1 | 12.8 | 40.0 | 50.5 | 22.4 |
| ΔKOOS Symptoms | 173 | 41.3 | 28.3 | 26.5 | 1.6 | 32.9 | 37.1 | 19.4 |
| ΔKOOS ADL | 170 | 50.0 | 36.7 | 29.4 | 20.2 | 36.5 | 45.6 | 27.2 |
| ΔKOOS Sports | 172 | 50.0 | 28.9 | 12.3 | 16.0 | 35.0 | 43.3 | 17.7 |
| ΔKOOS QoL | 173 | 65.3 | 44.2 | 23.1 | 20.0 | 47.5 | 58.6 | 26.3 |
|  |  |  |  |  |  |  |  |  |

^1^ Number of anchor responses that have at least one valid observation among the PROM scores/changes listed and evaluated after. ^2^ Response categories were presented in reverse order. Abbreviations: KOOS, Knee injury and Osteoarthritis Outcome Score; PROM, Patient Reported Outcome Measure; ADL, Activities of Daily Living; QoL, Quality of Life; VAS, Visual analogue scale; Δ (delta), “change in”.

Table S3. Spearman correlations between anchor and change in PROM score.

| **PROM** | **Spearman correlation coefficients** | | | | | |
| --- | --- | --- | --- | --- | --- | --- |
|  | *Anchor 1*  *Operation result* | Anchor 2  Change in joint | *Anchor 3*  *General health* | Anchor 4  Change in general health | *Anchor 5*  *Joint pain* | *Anchor 6**  *Joint satisfaction* |
| **HIP** | | | | | | |
| HOOS Pain | *-0.398* | **-0.382** | *-0.164* | -0.225 | *-0.485* | *0.439* |
| HOOS Symptoms | *-0.389* | **-0.358** | *-0.238* | -0.328 | *-0.390* | *0.377* |
| HOOS ADL | *-0.363* | **-0.332** | *-0.207* | -0.291 | *-0.439* | *0.381* |
| HOOS Sports | *-0.531* | -0.322 | *-0.396* | **-0.370** | *-0.365* | *0.402* |
| HOOS QoL | *-0.534* | **-0.467** | *-0.217* | -0.254 | *-0.576* | *0.442* |
| **KNEE** | | | | | | |
| KOOS Pain | *-0.505* | **-0.443** | *-0.160* | -0.288 | *-0.530* | *0.458* |
| KOOS Symptoms | *-0.379* | **-0.292** | *-0.054* | -0.225 | *-0.438* | *0.397* |
| KOOS ADL | *-0.425* | **-0.343** | *-0.160* | -0.236 | *-0.422* | *0.433* |
| KOOS Sports | *-0.409* | **-0.335** | *-0.321* | -0.299 | *-0.440* | *0.433* |
| KOOS QoL | *-0.638* | **-0.485** | *-0.390* | -0.385 | *-0.608* | *0.579* |

Anchors that are not retrospective transition anchors indicated in italics. Anchor chosen for estimation of Minimal clinically important improvement (MCII) indicated in bold. The wording of each anchor reported in this table is in abridged form. * For Anchor 6 the response categories were presented in reverse order vis a vis the other anchors, see Tables S1 and S2. Abbreviations: HOOS, Hip disability and Osteoarthritis Outcome Score; KOOS, Knee injury and Osteoarthritis Outcome Score; PROM, Patient Reported Outcome Measure; ADL, Activities of Daily Living; QoL, Quality of Life.

Table S4. Spearman correlations between anchor and PROM score.

| **PROM** | **Spearman correlation coefficients** | | | | | |
| --- | --- | --- | --- | --- | --- | --- |
|  | Anchor 1  Operation result | *Anchor 2*  *Change in joint* | Anchor 3  General health | *Anchor 4*  *Change in general health* | Anchor 5  Joint pain | Anchor 6*  Joint satisfaction |
| **HIP** | | | | | | |
| HOOS Pain | -0.522 | *-0.420* | -0.312 | *-0.288* | **-0.656** | 0.495 |
| HOOS Symptoms | -0.485 | *-0.373* | -0.305 | *-0.310* | **-0.531** | 0.448 |
| HOOS ADL | -0.492 | *-0.369* | -0.431 | *-0.376* | **-0.500** | 0.452 |
| HOOS Sports | **-0.492** | *-0.330* | -0.472 | *-0.408* | -0.342 | 0.397 |
| HOOS QoL | -0.566 | *-0.471* | -0.338 | *-0.306* | **-0.603** | 0.459 |
| EQ-5D Index | -0.414 | *-0.411* | **-0.563** | *-0.493* | -0.453 | 0.389 |
| EQ-VAS | -0.310 | *-0.241* | **-0.649** | *-0.403* | -0.217 | 0.236 |
| **KNEE** | | | | | | |
| KOOS Pain | -0.639 | *-0.511* | -0.349 | *-0.307* | **-0.775** | 0.600 |
| KOOS Symptoms | -0.500 | *-0.439* | -0.210 | *-0.272* | **-0.619** | 0.529 |
| KOOS ADL | -0.588 | *-0.473* | -0.439 | *-0.281* | **-0.630** | 0.623 |
| KOOS Sports | -0.466 | *-0.391* | -0.380 | *-0.314* | **-0.467** | 0.498 |
| KOOS QoL | -0.648 | *-0.512* | -0.432 | *-0.363* | **-0.697** | 0.652 |
| EQ-5D Index | -0.495 | *-0.433* | -0.537 | *-0.462* | **-0.554** | 0.577 |
| EQ-VAS | -0.346 | *-0.277* | **-0.602** | *-0.481* | -0.301 | 0.383 |

Anchors that are transition anchors indicated in italics. Anchor chosen for estimation of Patient-acceptable symptoms state (PASS) indicated in bold. for retrospective transition anchor. Bold for chosen anchor. The wording of each anchor reported in this table is in abridged form. * For Anchor 6 the response categories were presented in reverse order vis a vis the other anchors, see Tables S1 and S2. Abbreviations: HOOS, Hip disability and Osteoarthritis Outcome Score; KOOS, Knee injury and Osteoarthritis Outcome Score; PROM, Patient Reported Outcome Measure; ADL, Activities of Daily Living; QoL, Quality of Life; VAS, Visual analogue scale.

Table S5. Total in-sample misclassification (%) for different estimates of Minimal clinically important improvement (MCII) cut-offs for HOOS one year following hip arthroplasty, with gold standard given by acceptable/non-acceptable changes on the anchor used.

|  | **75^th^ percentile approach** | **Mean approach** | **ROC approaches** | | | |
| --- | --- | --- | --- | --- | --- | --- |
|  |  |  | **80% specificity** | **-45° tangent line** | **Youden** | **Pythagoras** |
| HOOS Pain | 9% | 15% | 55% | 32% | 21% | 21% |
| HOOS Symptoms | 7% | 17% | 41% | 41% | 49% | 17% |
| HOOS ADL | 8% | 17% | 61% | 43% | 5% | 43% |
| HOOS Sports | 16% | 29% | 51% | 29% | 29% | 29% |
| HOOS QoL | 5% | 13% | 54% | 26% | 13% | 26% |

See Table 1 for classification of anchor categories and Table 3 for MCII estimates and for which anchor was used for each domain. Abbreviations: HOOS, Hip disability and Osteoarthritis Outcome Score; ROC, Receiver Operating Characteristics; PROM, Patient Reported Outcome Measure; ADL, Activities of Daily Living; QoL, Quality of Life.

Table S6. Total in-sample misclassification (%) for different estimates of Minimal clinically important improvement (MCII) cut-offs for KOOS domains one year following knee arthroplasty, with gold standard given by acceptable/non-acceptable changes on the anchor used.

|  | **75^th^ percentile approach** | **Mean approach** | **ROC approaches** | | | |
| --- | --- | --- | --- | --- | --- | --- |
|  |  |  | **80% specificity** | **-45° tangent line** | **Youden** | **Pythagoras** |
| KOOS Pain | 8% | 16% | 40% | 21% | 21% | 21% |
| KOOS Symptoms | 14% | 28% | 37% | 31% | 37% | 37% |
| KOOS ADL | 9% | 26% | 33% | 29% | 33% | 33% |
| KOOS Sports | 13% | 27% | 32% | 27% | 35% | 23% |
| KOOS QoL | 10% | 14% | 31% | 24% | 14% | 14% |

See Table 2 for classification of anchor categories and Table 4 for MCII estimates and for which anchor was used for each domain. Abbreviations: KOOS, Knee injury and Osteoarthritis Outcome Score; ROC, Receiver Operating Characteristics; PROM, Patient Reported Outcome Measure; ADL, Activities of Daily Living; QoL, Quality of Life.

Table S7. Total in-sample misclassification (%) for different estimates of Patient-acceptable symptoms state (PASS) cut-offs for HOOS domains and EQ-5D Index and VAS one year following hip arthroplasty, with gold standard given by acceptable/non-acceptable states for the anchor used.

|  | **75^th^ percentile approach** | **Mean approach** | **ROC approaches** | | | |
| --- | --- | --- | --- | --- | --- | --- |
|  |  |  | **80% specificity** | **-45° tangent line** | **Youden** | **Pythagoras** |
| HOOS Pain | 21% | 32% | 21% | 21% | 15% | 21% |
| HOOS Symptoms | 20% | 30% | 30% | 20% | 20% | 20% |
| HOOS ADL | 26% | 30% | 33% | 26% | 30% | 30% |
| HOOS Sports | 27% | 38% | 38% | 27% | 38% | 27% |
| HOOS QoL | 20% | 34% | 20% | 20% | 20% | 20% |
| EQ-5D Index | 29% | 38% | 40% | 34% | 29% | 29% |
| EQ-VAS | 26% | 41% | 34% | 26% | 34% | 34% |

See Table 1 for classification of anchor categories and Table 5 for PASS estimates and for which anchor was used for each domain. Abbreviations: HOOS, Hip disability and Osteoarthritis Outcome Score; ROC, Receiver Operating Characteristics; PROM, Patient Reported Outcome Measure; ADL, Activities of Daily Living; QoL, Quality of Life; VAS, Visual analogue scale.

Table S8. Total in-sample misclassification (%) for different estimates of Patient-acceptable symptoms state (PASS) cut-offs for KOOS domains and EQ-5D Index and VAS one year following knee arthroplasty, with gold standard given by acceptable/non-acceptable states for the anchor used.

|  | **75^th^ percentile approach** | **Mean approach** | **ROC approaches** | | | |
| --- | --- | --- | --- | --- | --- | --- |
|  |  |  | **80% specificity** | **-45° tangent line** | **Youden** | **Pythagoras** |
| KOOS Pain | 22% | 30% | 13% | 18% | 14% | 14% |
| KOOS Symptoms | 20% | 31% | 20% | 20% | 24% | 20% |
| KOOS ADL | 25% | 32% | 25% | 23% | 23% | 23% |
| KOOS Sports | 24% | 41% | 30% | 25% | 27% | 27% |
| KOOS QoL | 26% | 32% | 20% | 20% | 20% | 20% |
| EQ-5D Index | 26% | 33% | 31% | 26% | 22% | 22% |
| EQ-VAS | 27% | 40% | 33% | 28% | 40% | 27% |

See Table 2 for classification of anchor categories and Table 6 for PASS estimates and for which anchor was used for each domain. Abbreviations: HOOS, Hip disability and Osteoarthritis Outcome Score; ROC, Receiver Operating Characteristics; PROM, Patient Reported Outcome Measure; ADL, Activities of Daily Living; QoL, Quality of Life; VAS, Visual analogue scale.
